# Plain Language Summary

**DOI:** 10.2903/j.efsa.2023.p210401

**Published:** 2023-04-19

## Abstract

This publication is linked to the following EFSA Journal article: http://onlinelibrary.wiley.com/doi/10.2903/j.efsa.2023.6857/full

efsa JOURNAL

19 April, 2023

## Re-evaluation of the risks to public health related to the presence of bisphenol A (BPA) in foodstuffs

**Disclaimer**

- This plain language summary (PLS) is a simplified communication of EFSA's *Re-evaluation of the risks to public health related to the presence of bisphenol A (BPA) in foodstuffs*. The full EFSA report can be found here.
- The purpose of the PLS is to enhance transparency and inform interested parties on EFSA's work on the topic using simplified language to present a summary of the main findings.

### Background to the request to re-evaluate health risks of BPA in food

- EFSA is responsible for providing independent scientific advice on food and feed-related risks to EU risk managers and policy makers, and for communicating its advice to the public and stakeholders in the food chain (primary producers, consumer organisations, etc.).
- In 2015, EFSA established a safe level of BPA intake: a temporary tolerable daily intake (t-TDI) for BPA was set at 4 µg/kg body weight per day (4 µg is 4 micrograms or 4 millionths of a gram).
- The TDI was made temporary in 2015 as EFSA scientists identified several data gaps and uncertainties, which they committed to reassess when data became available, particularly data from a 2-year study from the US National Toxicology Program.
- This t-TDI was compared with the estimated dietary exposure to BPA and no health concerns were raised for consumers from any age group (including unborn children, infants and adolescents).
- The t-TDI was also compared with the estimated exposure to BPA from non-dietary sources (dust, toys, cosmetics and thermal paper) and low health concerns were raised.
- Given the uncertainties about some of the toxic effects of BPA identified by EFSA's scientists, the European Commission* requested that EFSA re-evaluate the risks to public health from BPA in foodstuffs.

*The European Commission and Member State representatives are responsible for setting limits on the amount of a chemical that could migrate from food packaging into food.

### What was EFSA asked to do?

- Develop a protocol detailing upfront the criteria for studies inclusion and appraisal to ensure an efficient and transparent re-evaluation of BPA safety in foodstuffs.
- Use the protocol to assess new data not previously evaluated by EFSA on the risks of BPA exposure.
- Address the uncertainties about BPA toxicity on mammary gland proliferation, and on developmental and reproductive, metabolic, neurobehavioural* and immune systems.
- Establish a TDI to replace the t-TDI set in 2015.

*The link between a person's neurological state and their behaviour

### How did EFSA carry out this work?

- The re-evaluation was conducted using a systematic approach.
- Government-appointed experts from Denmark, France, Germany, the Netherlands, Norway, Sweden and Switzerland, and independent scientists appointed by EFSA, developed the detailed protocol for the BPA re-evaluation.
- The protocol was published in December 2017 after being submitted to a public consultation and subsequently revised and updated.
- In September 2018, an EFSA working group of scientific experts was set to start the evaluation of scientific data that had not previously been assessed by the EFSA on possible adverse effects of BPA, in line with the protocol.
- The studies included in the re-evaluation were assessed for reliability and relevance and conclusions were based on all the scientific evidence combined.
- In November 2021, the Panel on Food Contact Materials, Enzymes and Processing Aids (CEP) endorsed EFSA's scientific draft opinion on the re-evaluation of the risks to public health related to the presence of bisphenol A (BPA) in foodstuffs and this draft was submitted to public consultation from December 2021 to February 2022.
- The comments received during the public consultation were discussed by the working group of scientific experts and the opinion was revised where considered necessary. The revised opinion was finally adopted by the CEP Panel in December 2022.

### What data were used?

- The re-evaluation was based on published evidence that had not previously been considered by EFSA.
- The literature search for the re-evaluation covered the period from 1 January 2013 to 15 October 2018.
- The literature search for evidence on genotoxicity was extended to 21 July 2021.
  During the evaluation process, EFSA also liaised with interested parties and/or stakeholders to discuss the draft opinion.

### What were the limitations/uncertainties?

- The re-evaluation included a large number of non-standard endpoints and studies.
- Uncertainties related to the identification and characterisation of the toxicological reference point from which to derive the TDI were taken into account by the CEP Panel by applying an additional uncertainty factor of 2 in calculating the new threshold.
- The newly derived TDI was compared with the dietary exposure estimates for BPA from 2015.
- Any changes in BPA dietary exposure since 2015 (e.g. due to new regulatory restrictions on the use of BPA) were not taken into account in the risk evaluation.

### What were the outcomes and their implications?

The outcomes and implications of EFSA's re-evaluation of risks from BPA in foodstuffs are as follows:

- EFSA established a new TDI of 0.2 ng/kg body weight per day (0.2 ng is 0.2 nanograms or 0.2 billionths of a gram).
- This new TDI is 20,000 times lower than the previous t-TDI of 4 µg/kg body weight per day.
- Estimated dietary exposure to BPA from 2015 EFSA opinion is two to three orders of magnitude higher than the new tolerable level of 0.2 ng/kg body weight per day.
- BPA levels far higher than the new TDI are a health concern for all age groups of the general population.
- EFSA's scientific advice on BPA will inform future discussions among EU lawmakers on the measures to take to protect consumers.

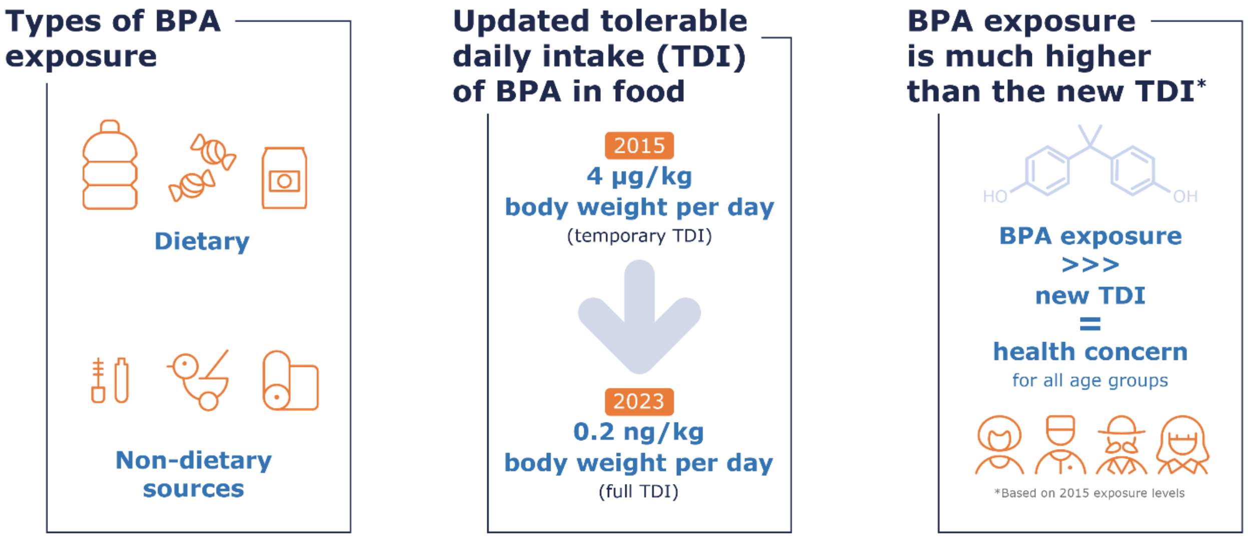

**Figure 1: BPA exposure** (information taken from *Re-evaluation of the risks to public health related to the presence of bisphenol A (BPA) in foodstuffs*)
